# The Development of Alginate/Ag NPs/Caffeic Acid Composite Membranes as Adsorbents for Water Purification

**DOI:** 10.3390/membranes13060591

**Published:** 2023-06-09

**Authors:** Angela Spoială, Cornelia-Ioana Ilie, Georgiana Dolete, Gabriela Petrișor, Roxana-Doina Trușcă, Ludmila Motelica, Denisa Ficai, Anton Ficai, Ovidiu-Cristian Oprea, Mara-Lia Dițu

**Affiliations:** 1 Department of Science and Engineering of Oxide Materials and Nanomaterials, Faculty of Chemical Engineering and Biotechnologies, University Politehnica of Bucharest, 1-7 Gh Polizu Street, 011061 Bucharest, Romania; angela.8317@gmail.com (A.S.);; 2 National Centre for Micro and Nanomaterials & National Centre for Food Safety, Faculty of Chemical Engineering and Biotechnologies, University Politehnica of Bucharest, Spl. Independentei 313, 060042 Bucharest, Romania; 3 Department of Inorganic Chemistry, Physical Chemistry and Electrochemistry, Faculty of Chemical Engineering and Biotechnologies, University Politehnica of Bucharest, 1-7 Gh Polizu Street, 050054 Bucharest, Romania; 4 Academy of Romanian Scientists, 3 Ilfov Street, 050045 Bucharest, Romania; 5 Faculty of Biology, University of Bucharest, 1-3 Aleea Portocalelor, 060101 Bucharest, Romania

**Keywords:** silver nanoparticles, alginate, caffeic acid, antibacterial, antifungal, antimicrobial, composite membranes, adsorbent, lead, water purification

## Abstract

Since the water pollution problem still affects the environmental system and human health, the need to develop innovative membranes has become imperious. Lately, researchers have focused on developing novel materials to help diminish the contamination problem. The aim of present research was to obtain innovative adsorbent composite membranes based on a biodegradable polymer, alginate, to remove toxic pollutants. Of all pollutants, lead was chosen due to its high toxicity. The composite membranes were successfully obtained through a direct casting method. The silver nanoparticles (Ag NPs) and caffeic acid (CA) from the composite membranes were kept at low concentrations, which proved enough to bestow antimicrobial activity to the alginate membrane. The obtained composite membranes were characterised by Fourier transform infrared spectroscopy and microscopy (FTIR), scanning electron microscopy (SEM), and thermogravimetric analysis (TG-DSC). Swelling behaviour, lead ion (Pb^2+^) removal capacity, regeneration and reusability were also determined. Further, the antimicrobial activity was tested against selected pathogenic strains (*S. aureus*, *E. faecalis* sp., *P. aeruginosa*, *E. coli* and *C. albicans*). The presence of Ag NPs and CA improves the antimicrobial activity of the newly developed membranes. Overall, the composite membranes are suitable for complex water treatment (removal of heavy metal ions and antimicrobial treatment).

## 1. Introduction

Rapid industrialisation worldwide has led to large production and improper discharge of heavy metals (HMs) within the environment, equally polluting land and water systems [1,2,3]. Toxic chemicals and HMs can potentially destroy the natural environment, becoming alarming when they accumulate through the food chain and affect human health [4,5,6,7]. Besides all HMs, lead (Pb) is one of the most serious and harmful pollutants [8,9]. Lead, the most toxic and non-biodegradable HM, is highly used in smelting, mining, battery manufacturing, painting and tanning [9,10,11,12].

Long-term accumulation or exposure to Pb may occur with significant health problems such as nervous, digestive, reproductive, immune and urinary systems [13,14,15,16,17]. Due to its high toxicity to human health, the World Health Organization (WHO) recommended not exceeding the highest contamination concentration of 0.010 mg/L in drinking water [18]. People are becoming interested in the importance of Pb toxicity on human health [19]. Therefore, scientists and researchers have focused on developing novel alternative materials to remove Pb from pollutant wastewater [20,21,22,23]. Up until now, traditional methods have been used to remove toxic pollutants. In addition, various technologies have been employed to remove Pb from wastewater, such as coagulation/flocculation, chemical precipitation, ion exchange, electrodeposition, ultrafiltration or adsorption [24,25,26]. Considering these techniques, adsorption is a promising technology for HM removal due to the following benefits: cost-efficiency, easy usability, eco-friendly and low energy consumption [27,28,29]. Recently, some studies focused on developing ceramic membranes for HM removal from wastewater. Results showed ceramic membranes are more effective in metal removal than polymer membranes [30,31].

Alginate was chosen as an adsorbent based on critical issues of environmental aspects and high adsorption performance after modification or cross-linking [32,33]. Alginate is a binary linear biopolymer considered the ideal eco-friendly material for developing adsorbent membranes for wastewater treatment. Choosing alginate biopolymers for developing adsorbent membranes was based on their biocompatibility, biodegradability and cost-effectiveness [34]. Besides its non-toxicity and good adsorbent capacity, it possesses plenty of hydroxyls (-OH) and carboxyl moieties (-COOH) on the polymer backbone, which serve in coordination and ion exchange [32,35].

Furthermore, the hydroxyl and carboxyl functional groups on its molecular chain could allow its GG blocks to cross-link with calcium chloride by exchanging Na^+^ with Ca^2+^. When the ion exchange occurs, an insoluble gel is formed with the characteristics of an “egg-box” structure [36]. This fact changes and improves the adsorption capacity of the adsorbent membranes. It has been proved that the alginate structure’s carboxyl functional groups are more effective in chelation than many others (hydroxyl, amine, amide, etc.) [35]. 

Numerous materials have been incorporated with alginate hydrogel to enhance the performance and stability of environmental applications. Many research studies have indicated that materials such as activated carbon, carbon nanotubes, graphene oxide, nanoparticles, magnetic materials and even microorganisms have been encapsulated in alginate for environmental applications [37,38]. Literature provides evidence regarding the synergism between alginate and the above materials by developing composite membranes, suggesting good potential for real-world applications [37,39,40,41].

Very recently, papers have shown that alginate-based membranes possess great nano-filtration performance towards cleaning properties. It has also been proven that adding diverse materials such as graphene oxide and carbon nanotubes improved the membrane stability and anti-fouling properties [42,43]. Other studies reported great results on the obtained alginate membranes specifying that they could be used as promising candidates for green organic solvent nanofiltration [44,45].

Papageorgious et al. [46] synthesised calcium-alginate beads from *Laminaria digitata* for the adsorption of Cu^2+^–Cd^2+^, Pb^2+^–Cd^2+^ and Pb^2+^–Cu^2+^ mixtures from aqueous metal solutions. Results confirmed that the formulated alginate beads are promising for HM adsorption from wastewater feeds [46]. Another interesting adsorbent is chitosan [47]. Some authors have developed an environment-friendly and effective alginate-chitosan hybrid adsorbent for the Pb^2+^ removal from water by the freeze-drying method [48].

Unfortunately, alginate does not exhibit antibacterial activity. Therefore, it cannot be used to kill water-borne pathogens. The possible use of nanoparticles as antimicrobial agents for water treatment is reported in the literature [49,50]. This shortcoming can be solved by loading the alginate membranes with antimicrobial agents like nanoparticles [51]. Ag NPs are one of the most effective antimicrobial agents capable of killing microorganisms, including viruses, bacteria and fungi [52]. Their dimensions strongly influence their antimicrobial activity. Silver nanoparticles have numerous applications in diverse fields. Besides its biomedical, engineering, electronics and consumer products applications, it has great potential in environmental remediation [53,54,55]. Another important substance of interest for loading within the adsorbent membranes to enhance its antibacterial activity is caffeic acid. Caffeic acid (3,4-hydroxycinnamic acid) belongs to the phenolic acids, an essential polyphenols class [56,57,58]. Among the many properties of caffeic acid, in addition to its antibacterial activity, other important biological activities include antioxidant, anti-inflammatory and immunomodulatory [59,60,61,62]. Caffeic acid is also a well-known chelating agent that can improve the membrane adsorption capacity [63,64].

In the current study, we report the synthesis of an alginate/Ag NPs/CA composite membrane for the first time through a simple casting method used for water treatment. Similar studies based on silver nanoparticle-alginate membranes demonstrated their potential for antibacterial applications, such as the biomedical and food packaging industry [65,66,67]. Here for the first time, we report the addition in the composite membrane of a second antimicrobial/chelating agent, caffeic acid. The adsorption capacity was tested on simulated polluted water (using Pb^2+^ as a model pollutant). The antimicrobial properties were determined against a broad range of pathogen microorganisms, indicating the advantage of the composite membranes in disinfecting the wastewater. Therefore, such membranes can be used for water purification treatment. The membranes were characterised by Fourier-transform infrared spectroscopy and microscopy (FTIR), scanning electron microscopy (SEM) and thermogravimetric and differential scanning calorimetry analysis (TG-DSC). The membrane was tested for polyphenol release, regeneration and reusability. Heavy metals (HM) ion removal capacity and antimicrobial activity were also determined.

## 2. Materials and Methods

### 2.1. Materials

The sodium alginate, nitrate salt of lead, calcium chloride dihydrate, caffeic acid and Ag NPs (<150 nm particle dimension) were acquired from Sigma-Aldrich Merck, Burlington, MA, USA. All chemicals were used without further purification. A stock solution, containing 1000 mg/L lead was prepared by dissolving Pb(NO_3_)_2_ in distilled water.

For the antimicrobial evaluation Nutrient Broth No. 2 (NB) Mueller–Hinton Broth (M–H), Sabouraud Glucose with chloramphenicol and agar acquired from Sigma-Aldrich (Darmstadt, Germany) were used. The microbial strains used in this study were obtained from the Microorganisms Collection of the Department of Microbiology, Faculty of Biology and Research Institute of the University of Bucharest.

### 2.2. Membrane Preparation

**Figure 1:**
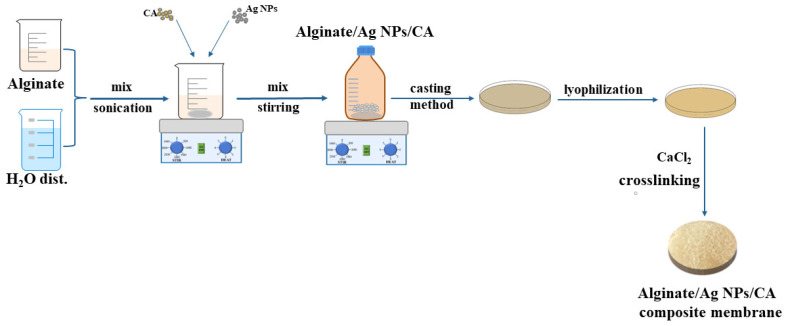
Synthesis of Alginate/ Ag NPs/CA composite membranes (CA-caffeic acid; Ag NPs-silver nanoparticles).

Figure 1 shows a graphic chart of alginate/Ag NPs/CA composite membranes preparation through a simple casting method similar to the study in [21]. First, sodium alginate (1.5 g) was dissolved, under magnetic stirring, in 50 mL of distilled water (at 25 °C for 24 h) to form a 3% solution.

Then, two solutions of 5 and 10 ppm silver nanoparticles were obtained. Next, caffeic acid (2, 10 and 20 ppm) was added to the previously obtained solutions. The final compositions of the obtained solutions are shown in Table 1 below.

Afterwards, the solutions were sonicated for 1 h and then left at 25 °C for 24 h while magnetically stirring to obtain homogenous solutions. Therefore, for this experiment, five solutions were cast in Petri dishes, lyophilised, cross-linked by immersion in 200 mL calcium chloride solution with 5% concentration for 10 min, and analysed using appropriate techniques.

### 2.3. Methods

The obtained composite membranes were characterised by FTIR spectroscopy and microscopy, SEM, TG-DSC, controlled release of polyphenolic, HM ion removal capacity, regeneration/reusability study and swelling capacity. Antimicrobial assessments were also performed.

The *Fourier transform infrared spectroscopy* (FTIR) spectra were recorded with a Nicolet iS50 FTIR spectrometer (Thermo Fisher Scientific, Waltham, MA, USA), using the attenuated total reflection accessory (ATR) (Thermo Fisher Scientific, Waltham, MA, USA). All spectra were obtained as the average of 32 scans in domain 400 and 4000 cm^−1^ at a resolution of 4 cm^−1^. *FTIR 2D maps* were obtained with a Nicolet iN10 MX FTIR microscope, equiped with a DTGS detector, between 600–4000 cm^−1^. Information about spatial distribution of the components was extracted from the 2D FTIR maps.

A Quanta Inspect F50 (FEI Company, Eindhoven, The Netherlands) was used to acquire the *scanning electron microscopy* (SEM) micrographs. The device is equipped with a field emission gun (FEG) and an energy dispersive X-ray spectrometer (EDX). Samples were covered with a 10 nm gold layer.

Thermal stability of the samples was determined with an STA 449 F3 Jupiter apparatus from Netzsch (Selb, Germany). Samples of ~10 mg from each membrane were placed in an open alumina crucible and heated with a speed of 10 °C min^−1^, up to 900 °C in oxidizing atmosphere (50 mL min^−1^ dried air).

A *high-performance liquid-chromatography*-type Agilent 1260 Infinity with Array Diode Detector (HPLC-DAD) was applied for analysing the controlled release of the polyphenol over time. The amount of bioactive substance in the samples and the calibration curve for caffeic acid in water were determined. The separation was done using an Aqua C18 column (250 × 4.6 mm, 5 μm) and a mobile phase composed of ultrapure water and methanol [59].

The *HM ion removal efficiencies* of the Alginate/Ag NPs/CA composite membranes were evaluated by measuring lead concentrations using inductively coupled plasma-mass spectrometry (ICP-MS). Prior to ICP-MS analysis, each membrane was exposed to 25 mL of 10, 50 and 100 mg/L lead solutions for 24 h. Lead nitrate solutions were prepared so that lead was present at the concentrations mentioned above to simulate the presence of HMs in polluting aqueous media with adverse effects on the environment. After exposure to the Pb(NO_3_)_2_ solutions, the samples were washed with distilled water to assess the Pb^2+^ absorbed in the membrane’s porous framework, removing traces from a stock solution. After washing, the membranes were placed in an oven at 40 °C until completely dried. Considering the dilutions made during the ICP-MS sample preparation and the dry mass of the sample, the final lead concentrations in the membranes were estimated. Finally, the removal efficiency (%R) of Pb^2+^ was calculated using Equation (1):

(1) $$\%R=\frac{C_{0}-C_{m}}{C_{0}}\cdot 10$$

where *C*_0_ is the maximum concentration of Pb that could have been adsorbed by the membranes based on the Pb(NO_3_)_2_ concentration of the solutions, and *C_m_* is the Pb concentration in the membranes determined by ICP-MS.

The *regeneration and reusability study* was also performed by ICP-MS analysis. Samples from each membrane (~140 mg) were exposed for three days to specific solutions. During the first day, each membrane was cross-linked with a 1% calcium chloride solution. A Pb^2+^ ion solution (10 mg/L) was used the next day to retain Pb^2+^ within the membranes. On the third day, two solutions of 1 and 10% calcium chloride were used to recover lead from the membrane. The samples (solutions and membranes) taken from the specific calcium chloride solutions were subjected to ICP-MS analysis. The recyclability of these adsorbent composite membranes is an essential aspect of energy consumption, economics and environmental protection issues.

Before ICP analysis, membranes for both retention and regeneration studies were weighed and subjected to digestion with 8 mL HNO_3_ in a microwave oven (Ethos UP, Milestone Inc., Sorisole, Italy), using a program for high organic content matrices (35 min at 200 °C, using a microwave power of 1800 W). After cooling, the samples were diluted with Milli-Q water up to 100 mL. Second, the samples were diluted 1000-fold to fit within the calibration range. Solutions for the regeneration study were diluted 10-fold. Measurements were performed with an Agilent 8800 Triple Quadrupole ICP-MS (Agilent Technologies, Tokyo, Japan), equipped with an ASX500 autosampler, Peltier cooling spray-chamber (2 °C), MicroMist concentric nebuliser, nickel sampler, skimmer cones and a 2.5 mm internal diameter torch. The equipment was tuned according to the manufacturer and calibrated with five calibration standards ranging from 0.5 to 10 µg/L Pb. ICP-MS’s operating conditions were the following: nebuliser pump set to 0.1 RPS, 1 L/min carrier gas flow, 0.7 mL/min He flows and 1550 W RF power. The lead calibration curve showed excellent linearity with a correlation coefficient of R^2^ = 0.9999.

The *adsorption/swelling capacity* was studied by immersing samples of ~2 cm × 2 cm in 0.2 L water. Each sample had its mass determined before starting the experiment and was weighted again at the following time intervals: 0.25, 0.5, 1, 2, 4, 6, 12 and 24 h. The equation 2 was used to calculate the swelling capacity:

(2) $$\mathrm{Swelling}\;\mathrm{capacity}\;\left(\%\right)=\frac{M_{h,t}{\;x\;M}_{i}}{M_{i}}\times 100,$$

M_i_ is the initial membrane mass and M_h,t_ is the membrane mass after immersion in water. The experiment was performed in triplicate.

The antimicrobial assays of the composite membranes were performed using *Staphylococcus aureus* ATCC 25923, *Enterococcus faecalis* VRE 2566, *Enterococcus faecalis* ATCC 29212, *Escherichia coli* ATCC 25922, *Pseudomonas aeruginosa* ATCC 27853 and *Candida albicans* ATCC 10231. Bacterial cell suspensions of 1.5 × 10^8^ CFU/mL and yeast suspensions of 3 × 10^8^ CFU/mL were obtained from fresh cultures.

The samples (1 cm × 1 cm) were sterilised by UV irradiation for 30 min on both sides to remove any possible contamination. The sterility of the antimicrobial assays was established by maintaining each type of membrane in media for 24 h at 37 °C. The broth media’s clarity indicates the samples’ sterility [68,69,70,71].

An adapted spot diffusion method was used for the qualitative antimicrobial assay [72,73]. Specific media for each strain was placed in Petri dishes, which were then seeded with inoculums. Samples of the same size, from each membrane were added at equal distances. The biologically active compounds (silver nanoparticles and caffeic acid) were performed as control solutions with the concentration specifics. After a 24 h incubation at 37 °C, bioactive compounds diffused on the media surface and their influence of each sample against the pathogenic strains was evaluated. By measuring the growth inhibition diameters the sensibility of strains was assessed.

The membranes’ anti-adherent capacity was made by establishing the colony-forming units/mL values (CFU/mL) by the method explained in the preceding studies [21,68,73], in line with M100-31st Edition Standard [71]. The viable colony formation was expressed as the mean of the total number of colonies ×1/D (D = decimal dilution, for which the number of total colonies was determined). The release of bio-active compounds into the liquid media was quantitatively assessed using the decimal microdilution method. 30 µL of the liquid media were taken after the samples were incubated in the presence of 10^7^ bacterial cell density. According to the previous method, decimal dilutions were accomplished to determine CFU/mL. The assessments were done in three independent experiments [21,68,73].

Antimicrobial assessments were statistically analysed via GraphPad Prism 9.5 by GraphPad Software, San Diego, CA, USA. We compared the capacity of selected strains to adhere to the surface of the membranes using analysis of variance (ANOVA) and Holm–Šídák’s as multiple comparison tests. A *p*-value < 0.05 is considered statistically significant.

## 3. Results and Discussion

### 3.1. FTIR Spectroscopy and Microscopy

#### 3.1.1. FTIR Spectroscopy

**Figure 2:**
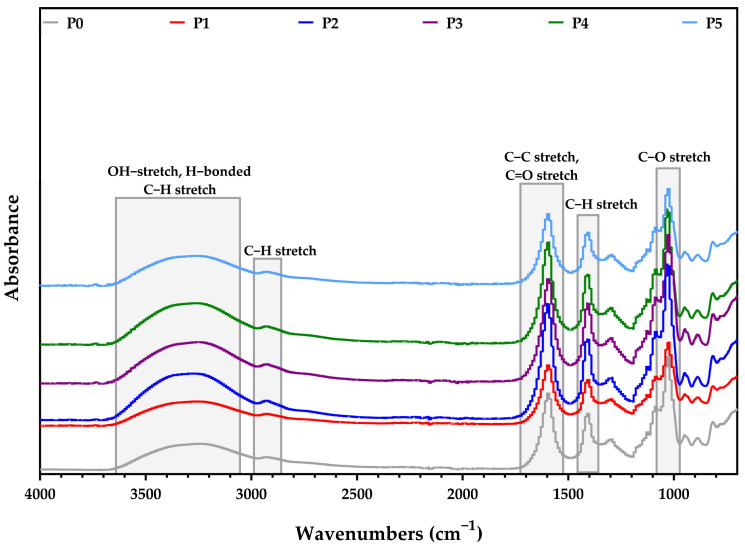
FTIR spectra for the composite membranes: P0-alginate, P1-Alg/Ag NPs1, P2-Alg/Ag NPs2, P3-Alg/Ag NPs/CA1, P4-Alg/Ag NPs/CA2, P5-Alg/Ag NPs/CA3.

The modifications that might occur within the composite membranes due to the Ag NPs and caffeic acid addition were investigated through FTIR spectroscopy. The most important assignments associated with the main adsorption peaks are presented in Figure 2 and also in Table 2.

The broad band from 3252–3272cm^−1^ was assigned to the vibration of the O-H bonds from alginate. The intense peaks between 2923–2930 cm^−1^ are assigned to the C-H asymmetric vibrations. The 1025–1031 cm^−1^ peaks corresponds to the glycosidic bond within the alginate structure. Small shifts could also be observed due to the interactions between the -COO^−^ group from the alginate molecule and the unreacted zone from Ag NPs. This information concords with data found in the literature [37,53,74].

#### 3.1.2. FTIR Microscopy

**Figure 3:**
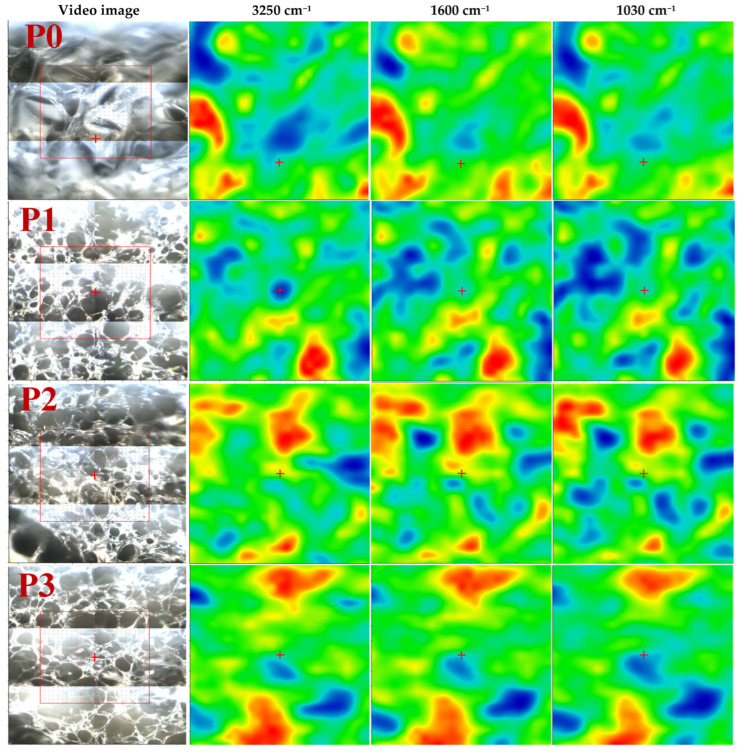

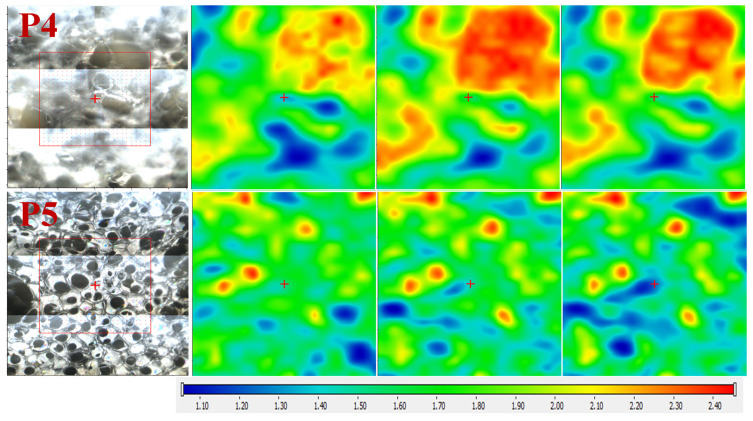
FTIR maps for the composite membranes: P0-alginate, P1-Alg/Ag NPs1, P2-Alg/Ag NPs2, P3-Alg/Ag NPs/CA1, P4-Alg/Ag NPs/CA2, P5-Alg/Ag NPs/CA3.

Through the help of FTIR microscopy, we could observe the spatial distribution of Ag NPs and CA within the polysaccharide matrix. Figure 3 illustrates the FTIR maps corresponding to the obtained membranes’ 3250 cm^−1^, 1600 cm^−1^ and 1030 cm^−1^, along with the microscopic view of the subjected region.

The FTIR maps, for all six samples, at these wavenumbers present striking similarities, which indicates a uniform distribution of components in the composite membranes. Therefore, the membranes are generally homogenous, meaning good Ag NPs and CA dispersion.

### 3.2. Scanning Electron Microscopy (SEM) Characterisation

The SEM analysis gives relevant data about surface morphology and other defects that might occur inside or on the surface of the membranes. 

**Figure 4:**
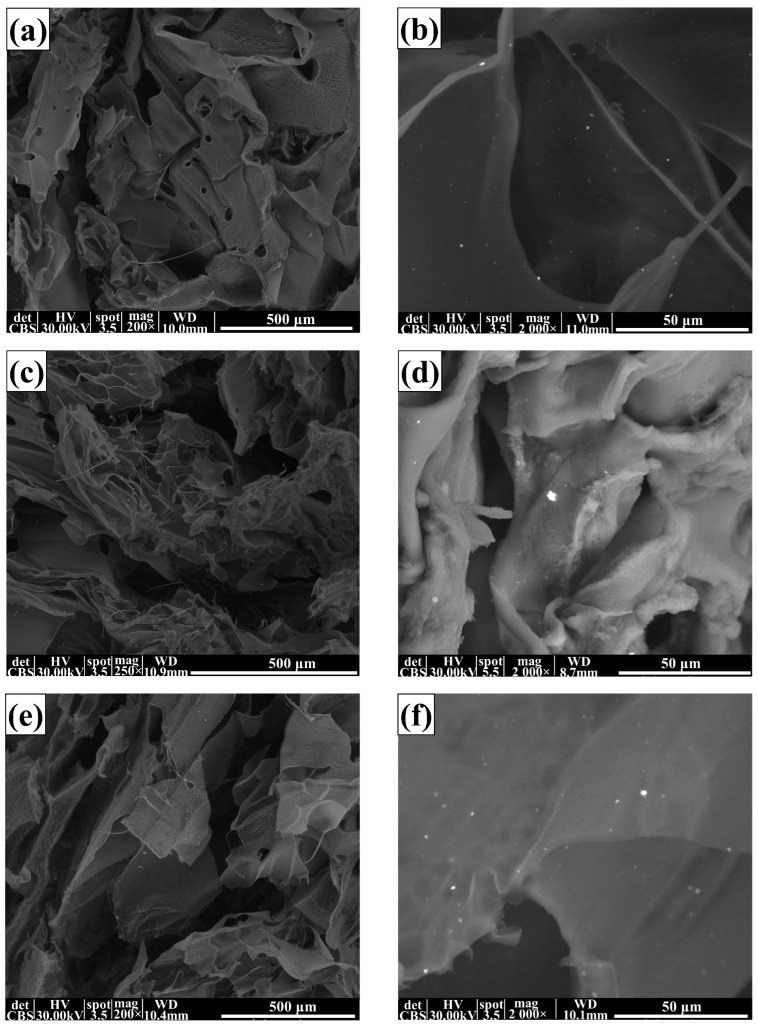

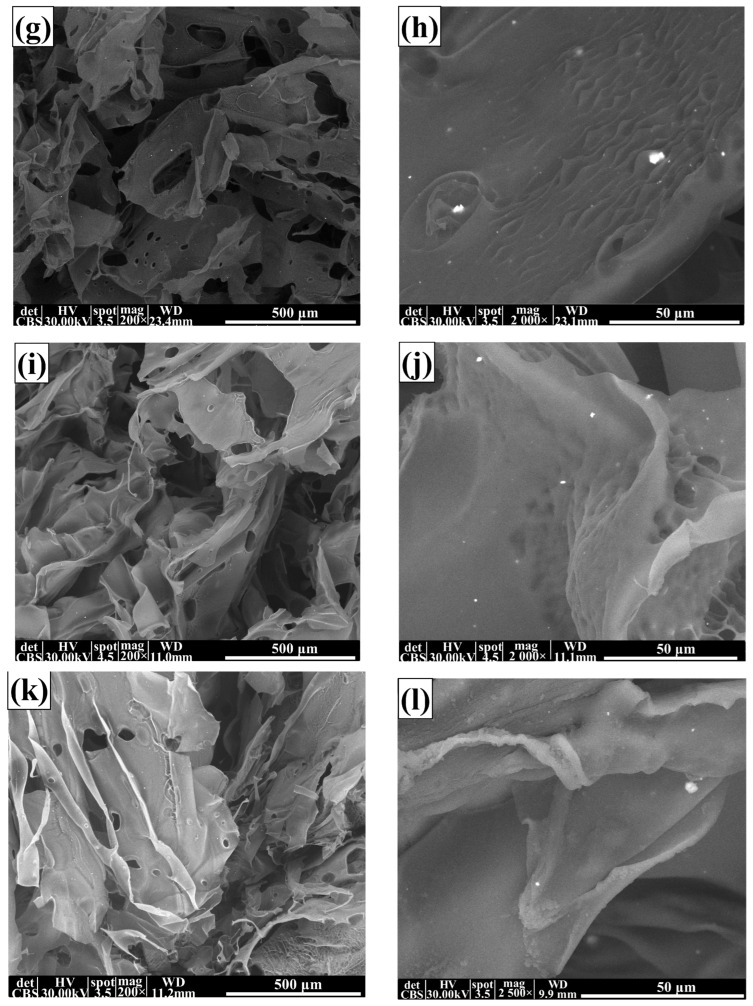
SEM images of alginate and alginate-based composite membranes for the composite membranes: P0-alginate (**a**,**b**), P1-Alg/Ag NPs1 (**c**,**d**), P2-Alg/Ag NPs2 (**e**,**f**), P3-Alg/Ag NPs/CA1 (**g**,**h**), P4-Alg/Ag NPs/CA2 (**i**,**j**), P5-Alg/Ag NPs/CA3 (**k**,**l**).

The morphology of the composite membranes is presented in Figure 4. The highly porous membrane with labyrinth system pores can be observed in all SEM images. Such pores result from the cross-linking processes when various shapes are obtained by fusing large flakes. The lyophilisation process similarly influences the formation of the smaller pores by forcing the water out of the structure. This resulted in a sponge-like appearance of the membrane, similar structures being reported in other literature studies [53,75]. In the SEM micrographs at higher magnification, Ag NPs (small white dots) could be observed on the samples’ surfaces.

**Figure 5:**
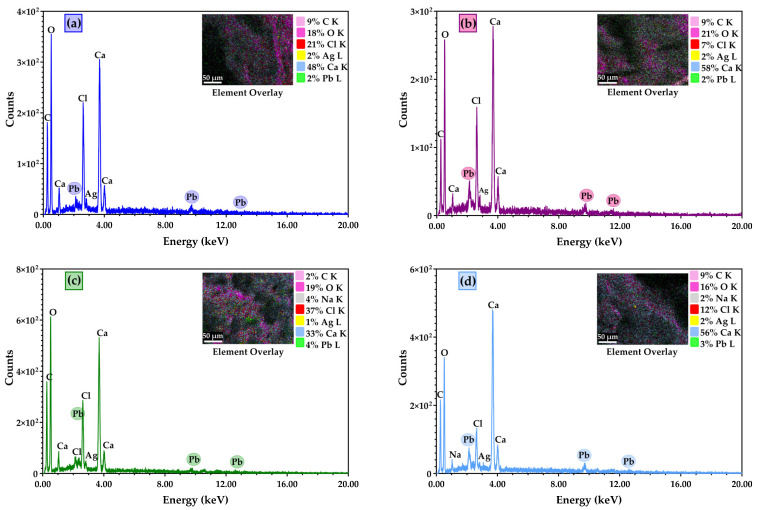
EDX spectra and elemental composition: (**a**) Alg/Ag NPs2, (**b**) Alg/Ag NPs/CA1, (**c**) Alg/Ag NPs/CA2, (**d**) Alg/Ag NPs/CA3.

EDX analysis indicates the elemental composition of the membranes obtained. EDX analysis was performed after the samples were cross-linked with calcium chloride and subjected to lead retention. The EDX results confirmed which samples retained lead solutions within their composition (Figure 5). The figure below shows which samples kept Pb within their pores.

### 3.3. Thermal (TG-DSC) Analysis

**Figure 6:**
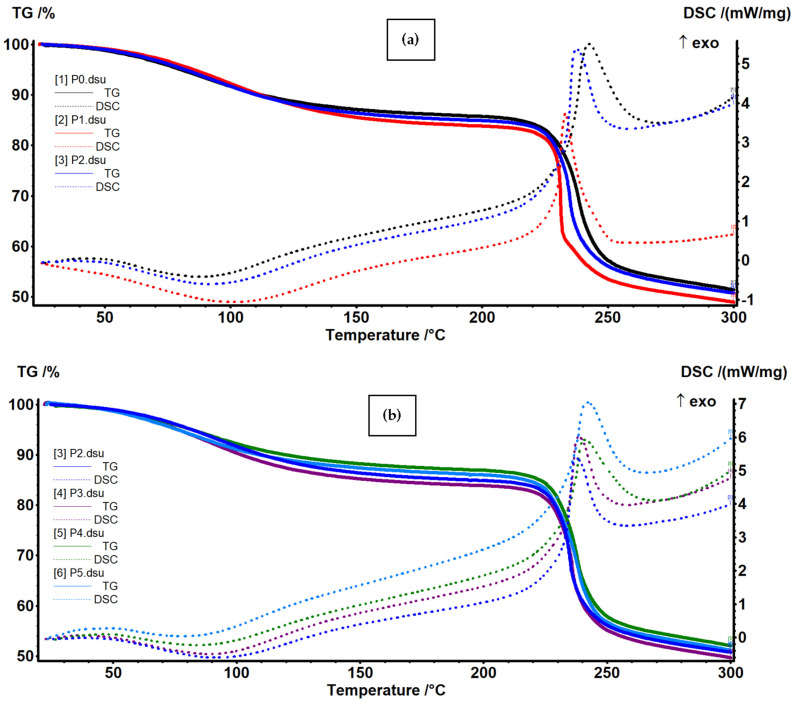
TG-DSC curves of simple alginate and alginate-based composite membranes: influence of silver nanoparticles—comparison of P0, P1 and P2 samples (**a**); influence of caffeic acid—comparison of P2, P3, P4 and P5 samples (**b**).

The thermal analysis data for simple alginate and alginate-based composite membranes are presented in Figure 6 for temperature intervals 20–300 °C and in Figures S1–S6 in detail. The samples are starting to lose residual water molecules up to 100 °C (~13.5% to ~16.5%), and a weak endothermic effect is associated to the process on the DSC curve [53]. The endothermic effect occurs at a higher temperature in the samples containing Ag NPs (101.3 and 92.2 °C for P1 and P2) when compared with the simple alginate one (87.7 °C for P0), indicating a minor improvement in thermal stability. Table 3 presents the principal data from thermal analysis.

Therefore, we can assign the first mass loss to the elimination of water-bound molecules from the alginate structure. Between 210–270 °C, the samples are losing 31–32% of their mass; this is associated with an exothermic effect, with a maximum between 233–243 °C. This indicates an oxidative process, most probably at secondary lateral moieties of the polymeric backbone [76]. The temperature onset for this process decreases from 231.2 °C for P0 to 224.9 and 230.9 °C for P1 and P2, indicating only minor changes induced by Ag NPs. After 270 °C, the samples lose mass continuously, up to ~530 °C, in a degradative–oxidative process of polymer fragmentation and burning. After 560 °C, the residual carbonaceous mass is burned in an exothermic process [77]. The residual mass represents 20–22%, and it consists of inorganic compounds (mainly CaO).

### 3.4. Controlled Release of Polyphenol

For the release study of polyphenols, only the samples with caffeic acid (P3, P4 and P5) were taken into consideration. For this experiment, the membrane samples were introduced in 50 mL distilled water and kept inside a thermoshaker at a controlled and monitored temperature of 37 °C to analyse the CA release. The dimensions of the membranes used for analysis were 40 × 20 × 5 mm, with a mass of ~0.56 g, which would result in a concentration of 15–147 ppm if the entire CA would have been released. The samples were analysed at 1, 2, 3, 4, 6, 8, 12, 36, 48, 60, 72, 75, 84 and 137 h. As a result of the study, a concentration higher than 10 ppm of caffeic acid was not determined in the solution. The CA was determined (Figure S6a) using a calibration curve between 10–250 ppm with an equation y = 9.0145x − 88.59 and R^2^ = 0.9988 (Figure S7b).

After the exposure time, the solutions were immediately injected into the HPLC. The obtained chromatograms of the tested samples (Alg/Ag NPs/ CA1, Alg/Ag NPs/ CA2 and Alg/Ag NPs/CA3) did not indicate the presence of CA (Figure S8a–c). In the samples collected up to 137 h, no interfering peaks related to caffeic acid were observed. This fact might be due to the cross-linked with calcium chloride, which blocked the membrane; therefore, no caffeic acid was released. This aspect is of great interest since caffeic acid has hydroxyl groups at positions 3 and 4, which will bond/interact with diverse functional groups of the other constituents of the composite membranes.

### 3.5. Removal Capacity of Lead Ions

The composite membranes were exposed for 24 h to 25 mL of 10, 50 and 100 mg/L lead nitrate concentration solution (these concentrations were obtained from a stock solution of 1000 mg/L) to assess the retention capacity of each obtained sample. The dimensions of the membrane samples used for analysis were 20 × 10 × 5 mm, with a mass of ~140 mg. The determinations of Pb ion quantity adsorbed onto the composite membranes were conducted through ICP-MS analysis.

**Figure 7:**
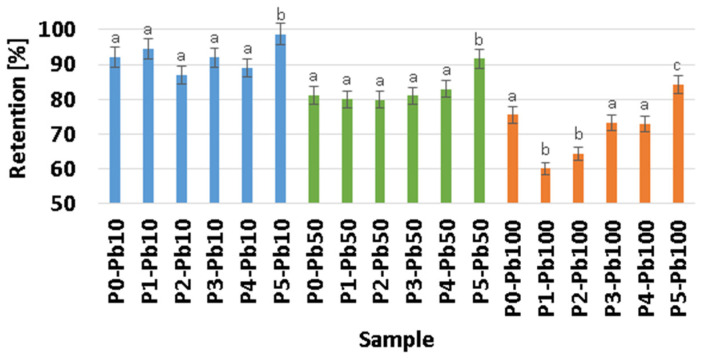
Per cent of lead absorption by P0–P5 composite membranes; function of concentration. Statistically significant differences between films (*p* < 0.05) are indicated by different small letters.

The absorption of Pb ions is due to the porous structure of the membranes, yielding promising results in terms of retention percentages, which ranged from 60.09 to 98.76%, as seen in Figure 7, with the highest values being obtained for the P5 sample in general.

**Figure 8:**
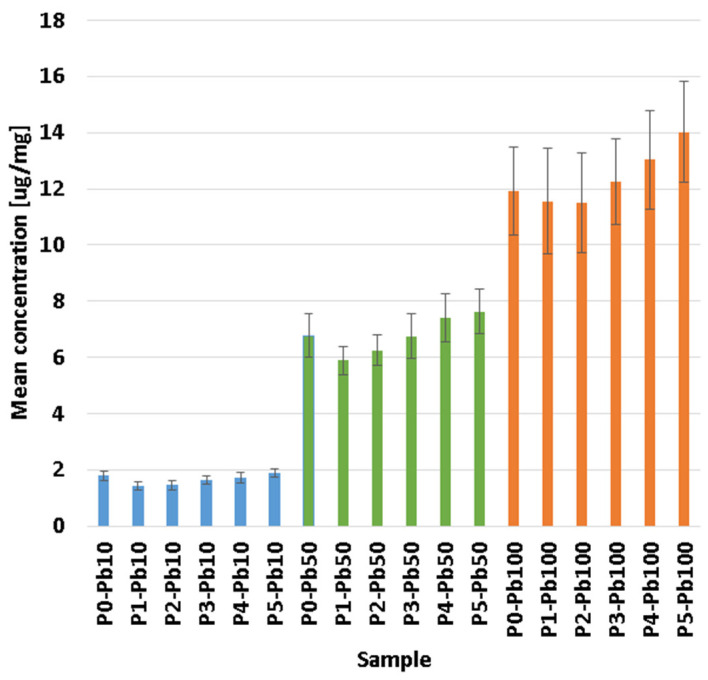
Lead absorption expressed as [µg/mg] by P0-P5 composite membranes, function of concentration.

One can see that the percentage of removed lead ions decreases as the Pb^2+^ concentration increases in the tested solutions. This is due to the saturation of the membrane with lead ions. Briefly, the membranes will remove a higher percentage of the existing lead ions when the solution is diluted and a smaller percentage when the solution is concentrated. To better understand membranes removal capacity, we factorised the membrane mass used in each experiment to see how much Pb^2+^ can be removed per membrane mg (Figure 8). It can be seen that although the removal percentage is higher in diluted solutions, the actual lead quantity removed is smaller. Consequently, the highest Pb^2+^ quantity removed can be observed in concentrated solutions, where, because of the saturation of the membrane, the calculated percentage is smaller.

Ag NPs and CA in the composite membranes have a mixed effect. While adding Ag NPs slightly reduces the adsorption capacity per mg, the presence of CA enhances the adsorption capacity due to multiple –OH moieties. Overall, the best removal capacity is exhibited by the P5 sample, with the highest CA content [78,79], but no statistical difference was observed.

### 3.6. Regeneration and Reusability Study

The recyclability and reusability of adsorbent membranes should be important, especially when involving energy consumption. Still, the most essential issue is environmental protection. Therefore, Table 4 illustrates the ICP-MS results of the solutions and composite membranes based on alginate, Ag NPs, and CA. Because we have used the 25 mL solution with a concentration of 10 mg/L Pb^2+^, the theoretical amount that can be adsorbed and removed is 250 µg Pb^2+^ [34,78].

Table 4 shows the results obtained for the membrane regeneration and reusability study. As expected, the membranes maintained in 10% CaCl_2_ can better remove lead from the membranes because the concentration of Ca ions is higher and can replace more Pb^2+^.

### 3.7. Sweeling/Adsorption Capacity

**Figure 9:**
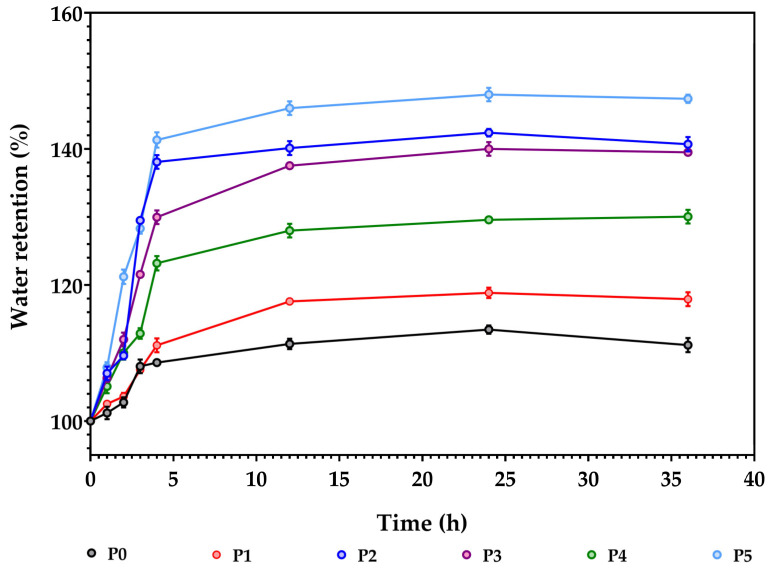
Swelling/adsorption capacity for the alginate and alginate-based composite membranes.

In the evaluation of the stability of the samples, the composite membranes were subjected to swelling measurements to determine the mass change during water immersion. The swelling behaviour was assessed in distilled water. Figure 9 presents the water uptake capacity on the synthesised composite membranes.

In the first 2–5 h, the samples absorb liquid, increasing their mass to saturation. Afterwards, further measurements indicated weight stabilisation, which could be associated with the maximum saturation limit. As can be viewed from Figure 9, the swelling capacity is highly influenced by the increased amount of Ag NPs and CA. This could be related to the pores’ existence within the membranes’ structure, which can induce a higher water retention capacity within the samples P1, P2, P3 and P4. The highest value was on P5 (~150%). Sample P0 contains only simple alginate, which exhibits a smaller water retention capacity (~110%). Literature confirms these results on swelling assays with similar polysaccharide membranes [53,80].

### 3.8. The Antimicrobial Assessments

#### 3.8.1. Qualitative Evaluation of the Antimicrobial Activity

**Figure 10:**
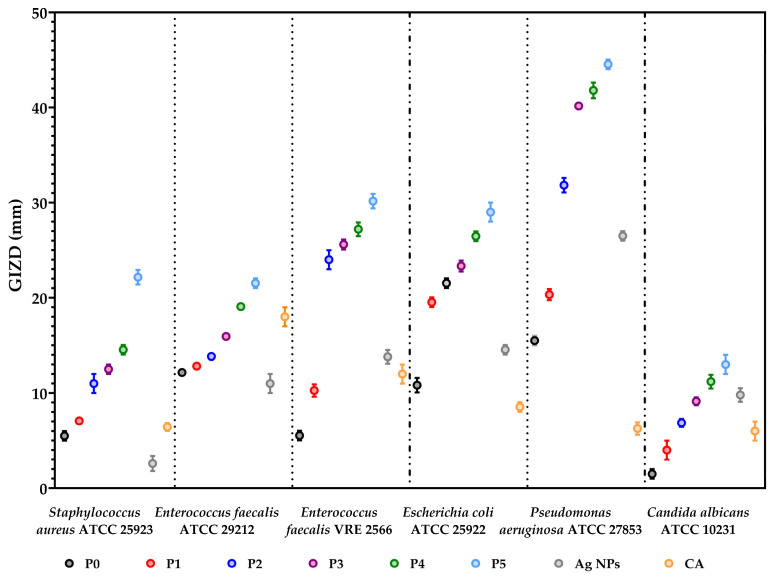
The graphical chart of GIZD. The significant impact of the samples on each microbial strain was statistically analysed by one-way ANOVA and Holm–Šídák’s multiple comparisons tests. The resulting data were statistically significant (*p* < 0.0001).

Qualitative evaluation of antimicrobial activity was made by measuring the growth inhibition zone diameters (GIZD). The data results are expressed in Figure 10 as mean values with standard deviation.

Figure 10 shows that all samples exhibited antimicrobial activity against the tested strains. The Gram-negative bacteria were most sensitive to the action of the alginate-based membranes. The membranes with silver NPs and CA (P3-P5) in composition determined the most significant sensitivity to *P. aeruginosa* ATCC 27853 than bioactive compounds. The inhibitory effect against Gram-negative and Gram-positive bacteria is evident in the case of membranes with a concentration of at least 0.66% silver NPs. The most sensitive strains were *P. aeruginosa* and *E. faecalis* sp. Alginate-based membranes determined a moderate inhibitory effect against *C. albicans*.

Inducing antimicrobial activity to alginate composite by adding silver nanoparticles is one of the logical choices [81,82,83]. A similar study [84] reported the antimicrobial activity of alginate-silver nanocomposite films against several pathogenic strains (*S. aureus*, *Salmonella typhimurium* and *Clostridium perfringens*). The 5–10 mg Ag films exhibited growth inhibition zone diameters of 10–12 mm. Susilowati et al. [85] presented that the antibacterial activity was affected by the concentration of Ag NPs, and *S. aureus* was more sensitive than *E. coli* to the action of alginate-silver films.

#### 3.8.2. Quantitative Evaluation of the Anti-Adherence Capacity of the Membranes

**Figure 11:**
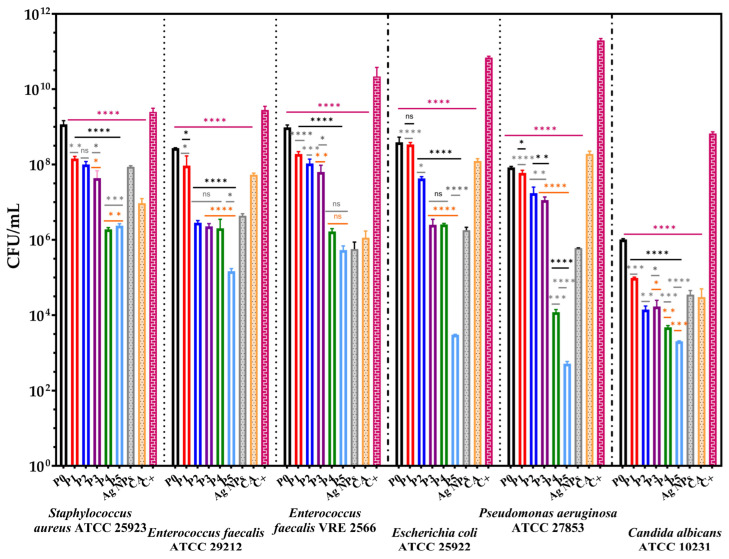
Graphical representation of CFU/mL values of tested strains to evaluate the release of bioactive compounds from alginate-based films into the broth media after 24 h. The data results were compared using one-way ANOVA and Holm–Šídák’s multiple comparisons tests (ns–not significant; * *p* < 0.03; ** *p* < 0.009; *** *p* < 0.0003; **** *p* < 0.0001).

**Figure 12:**
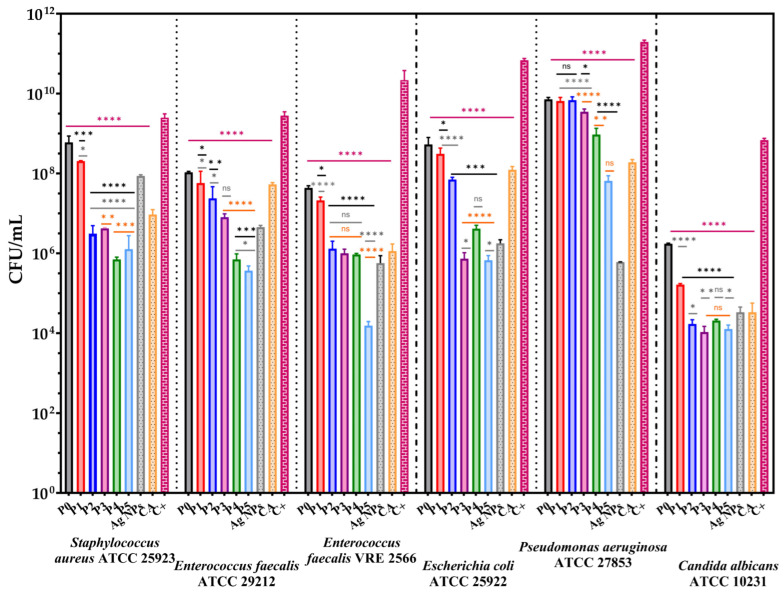
The anti-adherence capacity of the membranes against the tested strains. The data results were compared using one-way ANOVA and Holm–Šídák’s multiple comparisons tests (ns—not significant; * *p* < 0.04; ** *p* < 0.008; *** *p* < 0.0008; **** *p* < 0.0001).

According to the WHO, drinking water should be microorganism-free and not pose health risks to the human population [86]. Consequently, water quality from drinking water systems influences microbial biofilm formation. Moreover, the biofilm-forming capacity of microorganisms promotes even the attachment of other pathogens [87]. The influence of alginate-based membranes on selected strains’ growth and adherence capacity is presented in Figure 11 and Figure 12.

Figure 11 confirms the qualitative results and presents the inhibitory effect of bioactive compounds from the alginate-based membranes. The samples showed a decrease of viable colonies forming at most seven logarithmic units, which suggests the ability of the membranes to inhibit biofilm development. 

The alginate-based membranes enriched with CA and Ag NPs present a significant antimicrobial activity against the Gram-negative bacteria tested and *C. albicans* compared with the cell growth control. Moreover, it can be observed that enriching the alginate-based membranes with 1.31% caffeic acid decreased cell growth in liquid media by at least 4 CFU/mL logarithmic units. 

Quantitative results suggest a release and inhibitory effect of bioactive compounds from membranes after 24 h since they were in contact with selected strains in liquid broth.

Figure 12 presents the significant decrease in the CFU/mL values of the alginate-based membranes, suggesting the ability to inhibit the adherence of the bacteria cells on their surface. The capacity of the membranes to reduce cell proliferation in the liquid media was more pronounced in the case of Gram-positive bacteria. *S. aureus* was the most sensitive at concentrations above 0.66% Ag NPs. The membranes with 0.66 and 1.31% CA (P4 and P5) determined the most significant inhibitory effect compared to controls. This also can be observed for *E. faecalis* ATCC 29212. Moreover, in the case of P5, it can be remarked a significant decrease in CFU/mL values and a synergic effect between the CA and Ag NPs for all strains except *P. aeruginosa*.

In contrast with the qualitative results, membranes determined a moderate bacteriostatic effect against *P. aeruginosa* in liquid media. The growth rate for *C. albicans* decreased significantly (with 4 CFU/mL units) upon contact with an alginate-based membrane; still, substantial differences between the enriched samples with bioactive compounds cannot be observed. 

Overall, according to Figure 11 and Figure 12, the enrichment of the alginate membrane with Ag NPs and CA improves the antimicrobial activity of the newly developed membranes. Ag NPs inhibit bacterial growth by attaching to cell walls through electrostatic attraction, disrupting cell permeability by generating reactive oxygen species and DNA damage [88,89]. Caffeic acid also reduces biofilm development by increasing cell membrane permeability, depolarising and damaging cell membrane, inhibiting proline dehydrogenase production and cytosolic dehydrogenases activity, secretion of α-hemolysin, disrupting ATP synthesis, leakage of intracellular components etc. [90,91]. Furthermore, similar studies reported the pronounced inhibitory effect on bacterial growth, simultaneously with the increasing concentration of Ag NPs in alginate films [92,93,94].

## 4. Conclusions

Composite membranes based on alginate biopolymers and antibacterial agents, such as silver and caffeic acid, to develop adsorbent materials for removing lead ions from water systems were successfully synthesised. The Alginate/Ag NPs/ CA composite membranes were obtained through a simple casting method. The FTIR analysis indicates the presence of the main important functional groups found within the composite membranes. The SEM analysis reveals essential information on surface morphology, showing a highly porous-like appearance with labyrinth system pores. EDX analysis confirmed the elemental composition of the tested samples, indicating which samples had adsorbed Pb within their pores. The thermal analysis data indicates that samples had similar thermal behaviour, suggesting similar interactions in the polymer structure. The ICP-MS results confirmed the SEM and swelling/adsorbent test about the membranes**’** porosity, showing good Pb ions**’** absorbance. The removal capacity test on the obtained composite membranes showed a retention of 60.09 to 98.76%, which implies that Pb was adsorbed on the surface of the membranes. The regeneration and reusability study showed that the composite membranes kept in 10% CaCl_2_ had a better ability to remove Pb^2+^ due to the higher concentration of Ca^2+^. Further, the composite membranes were tested against selected pathogenic strains (*S. aureus*, *E. faecalis* sp., *P. aeruginosa*, *E. coli* and *C. albicans*). The qualitative evaluation, which measures the inhibition zone diameter around the samples, showed that all samples demonstrated antimicrobial activity against tested strains. Still, Gram-negative bacteria were more sensitive to the action of the alginate-based composite membranes. The quantitative evaluation of the anti-adherence capacity suggested the ability of the tested composite membranes to inhibit biofilm formation. In addition, the antimicrobial assessments of the subjected samples showed that adding Ag NPs and CA within the sample’s composition improves the antimicrobial activity of the newly developed adsorbent composite membranes. In conclusion, the results of this study show that these composite membranes are suitable for using them as adsorbent materials in water purification applications.

## Figures and Tables

**Table 1:** The alginate and alginate-based composite membranes composition.

Code	Sample Name	Alginate (g)	Ag NPs (mg)	Ag NPs (%)	Caffeic Acid (mg)	Caffeic Acid (%)	Water (mL)
P0	Alg 3%	1.5	0	0	0	0	50
P1	Alg/Ag NPs1	1.5	5	0.33	0	0	50
P2	Alg/Ag NPs2	1.5	10	0.66	0	0	50
P3	Alg/Ag NPs/CA1	1.5	5	0.33	2	0.13	50
P4	Alg/Ag NPs/CA2	1.5	5	0.33	10	0.66	50
P5	Alg/Ag NPs/CA3	1.5	5	0.33	20	1.31	50

**Table 2:** Assignment of relevant IR adsorption bands of alginate and alginate-based composite membranes.

Sample/Assignment	P0	P1	P2	P3	P4	P5
O-H and H-bonded (alcohols, phenols)	3253	3252	3272	3253	3260	3254
C-H stretch (aromatic)	-	-	-	3050	3054	3055
C-H stretch (alkanes)	2923	2927	2927	2930	2925	2925
C=O stretch	-	-	-	1640	1645	1645
C-C stretch	1593	1596	1596	1592	1596	1595
C-O stretch (alcohols, carboxylic acids, esters, ethers)	1031	1027	1027	1025	1028	1027

**Table 3:** Principal data from the thermal analysis (mass loss intervals and residual mass).

Sample	RT-210 °C	210–270 °C	270–360 °C	360–600 °C	Residual Mass
P0	14.72%	31.41%	8.14%	20.38%	20.61%
P1	16.54%	32.33%	6.07%	19.89%	21.41%
P2	15.47%	31.43%	7.17%	20.72%	21.93%
P3	16.46%	31.42%	7.72%	20.05%	20.52%
P4	13.54%	31.85%	16.96%	11.39%	20.10%
P5	14.39%	31.85%	18.72%	9.90%	20.00%

**Table 4:** ICP-MS results of the solutions and membranes after immersion in CaCl_2_ 1% and CaCl_2_ 10%.

Sample	CaCl_2_ 1%		CaCl_2_ 10%	
	Amount Pb in Solution [µg]	Amount Pb in Membrane [µg]	Amount Pb in Solution [µg]	Amount Pb in Membrane [µg]
P1	103.42 ± 0.28	133.01 ± 12.80	195.71 ± 1.73	41.05 ± 13.20
P2	98.09 ± 0.26	120.12 ± 13.30	187.62 ± 1.66	31.01 ± 2.90
P3	102.59 ± 0.10	128.11 ± 32.40	201.01 ± 1.63	29.12 ± 11.70
P4	108.37 ± 0.15	114.03 ± 14.40	211.57 ± 1.59	11.21 ± 10.30
P5	115.48 ± 0.03	131.05 ± 12.40	227.65 ± 1.41	19.02 ± 7.60

## Data Availability

Not applicable.

## Supplementary Materials

The following supporting information can be downloaded at: https://www.mdpi.com/article/10.3390/membranes13060591/s1, Figure S1: TG-DSC curves of P0 alginate membrane; Figure S2: TG-DSC curves of P1 alginate-based composite membrane; Figure S3: TG-DSC curves of P2 alginate-based composite membrane; Figure S4: TG-DSC curves of P3 alginate-based composite membrane; Figure S5: TG-DSC curves of P4 alginate-based composite membrane; Figure S6: TG-DSC curves of P5 alginate-based composite membrane; Figure S7: HPLC chromatogram of caffeic acid standards (a) and calibration curve (b); Figure S8: HPLC chromatograms for samples collected after 137h: (a) P3; (b) P4; (c) P5.

## References

[1] Zare E.N., Motahari A., Sillanpaa M. (2018). Nanoadsorbents based on conducting polymer nanocomposites with main focus on polyaniline and its derivatives for removal of heavy metal ions/dyes: A review. Environ. Res..

[2] Huang Z., Huang Z., Feng L., Luo X., Wu P., Cui L., Mao X. (2018). Modified cellulose by polyethyleneimine and ethylenediamine with induced cu(ii) and pb(ii) adsorption potentialities. Carbohydr. Polym..

[3] Abdelwahab N.A., Al-Ashkar E.A., El-Ghaffar M.A.A. (2015). Preparation and characterization of eco-friendly poly(p-phenylenediamine) and its composite with chitosan for removal of copper ions from aqueous solutions. Trans. Nonferrous Met. Soc. China.

[4] Mihaly M., Comanescu A.F., Rogozea E.A., Meghea A. (2010). Nonionic microemulsion extraction of ni (ii) from wastewater. Mol. Cryst. Liq. Cryst..

[5] Bhaumik M., Agarwal S., Gupta V.K., Maity A. (2016). Enhanced removal of Cr(vi) from aqueous solutions using polypyrrole wrapped oxidized MWCNTS nanocomposites adsorbent. J. Colloid Interface Sci..

[6] Fu F., Wang Q. (2011). Removal of heavy metal ions from wastewaters: A review. J. Environ. Manag..

[7] Carolin C.F., Kumar P.S., Saravanan A., Joshiba G.J., Naushad M. (2017). Efficient techniques for the removal of toxic heavy metals from aquatic environment: A review. J. Environ. Chem. Eng..

[8] Shen C., Zhao Y., Li W., Yang Y., Liu R., Morgen D. (2019). Global profile of heavy metals and semimetals adsorption using drinking water treatment residual. Chem. Eng. J..

[9] Yin N., Wang K., Wang L., Li Z. (2016). Amino-functionalized mofs combining ceramic membrane ultrafiltration for Pb (ii) removal. Chem. Eng. J..

[10] Ali I., Peng C., Lin D., Saroj D.P., Naz I., Khan Z.M., Sultan M., Ali M. (2019). Encapsulated green magnetic nanoparticles for the removal of toxic Pb(2+) and Cd(2+) from water: Development, characterization and application. J. Environ. Manag..

[11] Tatli Seven P., Iflazoglu Mutlu S., Seven I., Arkali G., Ozer Kaya S., Kanmaz O.E. (2021). Protective role of yeast beta-glucan on lead acetate-induced hepatic and reproductive toxicity in rats. Environ. Sci. Pollut. Res. Int..

[12] Ahmed M.J.K., Ahmaruzzaman M. (2016). A review on potential usage of industrial waste materials for binding heavy metal ions from aqueous solutions. J. Water Process Eng..

[13] Fang Y., Lu L., Liang Y., Peng D., Aschner M., Jiang Y. (2021). Signal transduction associated with lead-induced neurological disorders: A review. Food Chem. Toxicol..

[14] Samuel M.S., Shah S.S., Bhattacharya J., Subramaniam K., Pradeep Singh N.D. (2018). Adsorption of Pb(ii) from aqueous solution using a magnetic chitosan/graphene oxide composite and its toxicity studies. Int. J. Biol. Macromol..

[15] Wells E.M., Liu Y., Rolle-McFarland D., Mostafaei F., Zheng W., Nie L.H. (2018). In vivo measurement of bone manganese and association with manual dexterity: A pilot study. Environ. Res..

[16] Meng H., Wang L., He J., Wang Z. (2016). The protective effect of gangliosides on lead (Pb)-induced neurotoxicity is mediated by autophagic pathways. Int. J. Environ. Res. Public Health.

[17] Agraz-Cibrian J.M., Delgado-Rizo V., Segura-Ortega J.E., Maldonado-Gomez H.A., Zambrano-Zaragoza J.F., Duran-Avelar M.J., Vibanco-Perez N., Fafutis-Morris M. (2018). Impaired neutrophil extracellular traps and inflammatory responses in the peritoneal fluid of patients with liver cirrhosis. Scand. J. Immunol..

[18] Ahmad R., Mirza A. (2018). Facile one pot green synthesis of chitosan-iron oxide (Cs-Fe_2_O_3_) nanocomposite: Removal of Pb(ii) and Cd(ii) from synthetic and industrial wastewater. J. Clean. Prod..

[19] Filipoiu D.C., Bungau S.G., Endres L., Negru P.A., Bungau A.F., Pasca B., Radu A.F., Tarce A.G., Bogdan M.A., Behl T. (2022). Characterization of the toxicological impact of heavy metals on human health in conjunction with modern analytical methods. Toxics.

[20] Bai C., Wang L., Zhu Z. (2020). Adsorption of Cr(iii) and Pb(ii) by graphene oxide/alginate hydrogel membrane: Characterization, adsorption kinetics, isotherm and thermodynamics studies. Int. J. Biol. Macromol..

[21] Spoiala A., Ilie C.I., Dolete G., Croitoru A.M., Surdu V.A., Trusca R.D., Motelica L., Oprea O.C., Ficai D., Ficai A. (2022). Preparation and characterization of chitosan/TiO(2) composite membranes as adsorbent materials for water purification. Membranes.

[22] Spoială A., Ilie C.-I., Dolete G., Trușcă R.-D., Motelica L., Oprea O.-C., Ficai D., Ficai A., Andronescu E., Dițu L.-M. (2022). The development of antimicrobial chitosan/zno nanocomposite membranes for water purification. Rev. Română De Mater. Rom. J. Mater..

[23] Ahmad R., Mirza A. (2017). Adsorption of Pb(ii) and Cu(ii) by alginate-au-mica bionanocomposite: Kinetic, isotherm and thermodynamic studies. Process Saf. Environ. Prot..

[24] Enache D.F., Vasile E., Simonescu C.M., Culita D., Vasile E., Oprea O., Pandele A.M., Razvan A., Dumitru F., Nechifor G. (2018). Schiff base-functionalized mesoporous silicas (MCM-41, HMS) as Pb(ii) adsorbents. Rsc Adv..

[25] Enache D.F., Vasile E., Simonescu C.M., Razvan A., Nicolescu A., Nechifor A.C., Oprea O., Patescu R.E., Onose C., Dumitru F. (2017). Cysteine-functionalized silica-coated magnetite nanoparticles as potential nano adsorbents. J. Solid State Chem..

[26] Culita D.C., Simonescu C.M., Dragne M., Stanica N., Munteanu C., Preda S., Oprea O. (2015). Effect of surfactant concentration on textural, morphological and magnetic properties of CoFe_2_O_4_ nanoparticles and evaluation of their adsorptive capacity for Pb(ii) ions. Ceram. Int..

[27] Croitoru A.M., Ficai A., Ficai D., Trusca R., Dolete G., Andronescu E., Turculet S.C. (2020). Chitosan/graphene oxide nanocomposite membranes as adsorbents with applications in water purification. Materials.

[28] Ganiyu S.A., Lateef S.A. (2021). Review of adsorptive desulfurization process: Overview of the non-carbonaceous materials, mechanism and synthesis strategies. Fuel.

[29] Kamar F.H., Nechifor A.C., Nechifor G., Al-Musawi T.J., Mohammed A.H. (2017). Aqueous phase biosorption of Pb(ii), Cu(ii), and Cd(ii) onto cabbage leaves powder. Int. J. Chem. React. Eng..

[30] Choi N.-C., Cho K.-H., Kim M.-S., Park S.-J., Lee C.-G. (2020). A hybrid ion-exchange fabric/ceramic membrane system to remove As(v), Zn(ii), and turbidity from wastewater. Appl. Sci..

[31] Yoon S., Cho K.-H., Kim M., Park S.-J., Lee C.-G., Choi N.-C. (2023). Selenium removal from aqueous solution using a low-cost functional ceramic membrane derived from waste cast iron. Water.

[32] Monier M., Abdel-Latif D.A., Mohammed H.A. (2015). Synthesis and characterization of uranyl ion-imprinted microspheres based on amidoximated modified alginate. Int. J. Biol. Macromol..

[33] Simonescu C.M., Mason T.J., Calinescu I., Lavric V., Vinatoru M., Melinescu A., Culita D.C. (2020). Ultrasound assisted preparation of calcium alginate beads to improve absorption of Pb + 2 from water. Ultrason. Sonochem..

[34] Mousa N.E., Simonescu C.M., Patescu R.E., Lavric V., Culita D.C. (2017). Regeneration of calcium alginate and chitosan coated calcium alginate sorbents to be reused for lead (ii) removal from aqueous solutions. Rev. Chim..

[35] Cordova B.M., Jacinto C.R., Alarcon H., Mejia I.M., Lopez R.C., de Oliveira Silva D., Cavalheiro E.T.G., Venancio T., Davalos J.Z., Valderrama A.C. (2018). Chemical modification of sodium alginate with thiosemicarbazide for the removal of Pb(ii) and Cd(ii) from aqueous solutions. Int. J. Biol. Macromol..

[36] Niculescu A.G., Grumezescu A.M. (2022). Applications of Chitosan-Alginate-Based Nanoparticles-An Up-to-Date Review. Nanomaterials.

[37] Wang B., Wan Y., Zheng Y., Lee X., Liu T., Yu Z., Huang J., Ok Y.S., Chen J., Gao B. (2018). Alginate-based composites for environmental applications: A critical review. Crit. Rev. Environ. Sci. Technol..

[38] Mihaly M., Lacatusu I., Enesca I.A., Meghea A. (2008). Hybride nanomaterials based on silica coated C-60 clusters obtained by microemulsion technique. Mol. Cryst. Liq. Cryst..

[39] Xiangliang P., Jianlong W., Daoyong Z. (2005). Biosorption of Pb(ii) by pleurotus ostreatus immobilized in calcium alginate gel. Process Biochem..

[40] Jiao C., Xiong J., Tao J., Xu S., Zhang D., Lin H., Chen Y. (2016). Sodium alginate/graphene oxide aerogel with enhanced strength-toughness and its heavy metal adsorption study. Int. J. Biol. Macromol..

[41] Bee A., Talbot D., Abramson S., Dupuis V. (2011). Magnetic alginate beads for Pb(ii) ions removal from wastewater. J. Colloid Interface Sci..

[42] Ugur Nigiz F. (2020). Graphene oxide-sodium alginate membrane for seawater desalination through pervaporation. Desalination.

[43] Aburabie J., Nassrullah H., Hashaikeh R. (2023). Fine-tuning of carbon nanostructures/alginate nanofiltration performance: Towards electrically-conductive and self-cleaning properties. Chemosphere.

[44] Aburabie J.H., Puspasari T., Peinemann K.-V. (2020). Alginate-based membranes: Paving the way for green organic solvent nanofiltration. J. Membr. Sci..

[45] Wang Y., He Y., Yan S., Yin X., Chen J. (2019). Development of alginate hydrogel modified multifunctional filtration membrane with robust anti-fouling property for efficient water purification. Colloids Surf. A: Physicochem. Eng. Asp..

[46] Papageorgiou S.K., Katsaros F.K., Kouvelos E.P., Kanellopoulos N.K. (2009). Prediction of binary adsorption isotherms of Cu(2+), Cd(2+) and Pb(2+) on calcium alginate beads from single adsorption data. J. Hazard. Mater..

[47] Radulescu M., Ficai D., Oprea O., Ficai A., Andronescu E., Holban A.M. (2015). Antimicrobial chitosan based formulations with impact on different biomedical applications. Curr. Pharm. Biotechnol..

[48] Wang Z., Jin P., Wang M., Wu G., Sun J., Zhang Y., Dong C., Wu A.G. (2018). Highly efficient removal of toxic Pb2+ from wastewater by an alginate-chitosan hybrid adsorbent. J. Chem. Technol. Biotechnol..

[49] Motelica L., Oprea O.C., Vasile B.S., Ficai A., Andronescu E., Ficai D., Holban A.M. (2023). Antibacterial activity of solvothermal obtained zno nanoparticles with different morphology and photocatalytic activity against a dye mixture: Methylene blue, rhodamine b and methyl orange. Int. J. Mol. Sci..

[50] Motelica L., Vasile B.S., Ficai A., Surdu A.V., Ficai D., Oprea O.C., Andronescu E., Jinga D.C., Holban A.M. (2022). Influence of the alcohols on the zno synthesis and its properties: The photocatalytic and antimicrobial activities. Pharmaceutics.

[51] Plotniece A., Sobolev A., Supuran C.T., Carta F., Bjoerkling F., Franzyk H., Yli-Kauhaluoma J., Augustyns K., Cos P., De Vooght L. (2023). Selected strategies to fight pathogenic bacteria. J. Enzym. Inhib. Med. Chem..

[52] Xu M.Q., Luo H.Y., Rong H.W., Wu S.H., Zheng Z.X., Chen B.Y. (2023). Calcium alginate gels-functionalized polyurethane foam decorated with silver nanoparticles as an antibacterial agent for point-of-use water disinfection. Int. J. Biol. Macromol..

[53] Motelica L., Ficai D., Oprea O.C., Ficai A., Ene V.L., Vasile B.S., Andronescu E., Holban A.M. (2021). Antibacterial biodegradable films based on alginate with silver nanoparticles and lemongrass essential oil-innovative packaging for cheese. Nanomaterials.

[54] Spoiala A., Ficai D., Ficai A., Craciun L., Titu M.A., Andronescu E. (2021). Towards synthesis-derived applications of silver nanoparticles. Adv. Mater. Tech. Env..

[55] Kukushkina E.A., Hossain S.I., Sportelli M.C., Ditaranto N., Picca R.A., Cioffi N. (2021). Ag-based synergistic antimicrobial composites. A critical review. Nanomaterials.

[56] Stavinskay O., Laguta I., Kuzema P., Skorochod I., Roy A., Kurdish I. (2022). Preparation of composite based on caffeic acid and fumed silica and evaluation of its antioxidant and antimicrobial properties Br. Chem. J. Mold..

[57] Zeren S., Sahin S., Sumnu G. (2022). Encapsulation of Caffeic Acid in Carob Bean Flour and Whey Protein-Based Nanofibers via Electrospinning. Foods.

[58] Kepa M., Miklasinska-Majdanik M., Wojtyczka R.D., Idzik D., Korzeniowski K., Smolen-Dzirba J., Wasik T.J. (2018). Antimicrobial potential of caffeic acid against staphylococcus aureus clinical strains. Biomed. Res. Int..

[59] Petrisor G., Motelica L., Ficai D., Trusca R.D., Surdu V.A., Voicu G., Oprea O.C., Ficai A., Andronescu E. (2022). New mesoporous silica materials loaded with polyphenols: Caffeic acid, ferulic acid and p-coumaric acid as dietary supplements for oral administration. Materials.

[60] Enaru B., Socaci S., Farcas A., Socaciu C., Danciu C., Stanila A., Diaconeasa Z. (2021). Novel delivery systems of polyphenols and their potential health benefits. Pharmaceuticals.

[61] Catauro M., Barrino F., Dal Poggetto G., Crescente G., Piccolella S., Pacifico S. (2020). New SiO_2_/caffeic acid hybrid materials: Synthesis, spectroscopic characterization, and bioactivity. Materials.

[62] Espindola K.M.M., Ferreira R.G., Narvaez L.E.M., Silva Rosario A.C.R., da Silva A.H.M., Silva A.G.B., Vieira A.P.O., Monteiro M.C. (2019). Chemical and pharmacological aspects of caffeic acid and its activity in hepatocarcinoma. Front. Oncol..

[63] Craioveanu M.G., Stoica L., Constantin C., Oprea O. (2020). Cr(iii)_aq_ separation by flotation with multipolar collector. Sep. Sci. Technol..

[64] Boilet L., Cornard J.P., Lapouge C. (2005). Determination of the chelating site preferentially involved in the complex of lead(ii) with caffeic acid: A spectroscopic and structural study. J. Phys. Chem. A.

[65] Sharma S., Sanpui P., Chattopadhyay A., Ghosh S.S. (2012). Fabrication of antibacterial silver nanoparticle—Sodium alginate–chitosan composite films. Rsc Adv..

[66] Shao Y., Wu C., Wu T., Yuan C., Chen S., Ding T., Ye X., Hu Y. (2018). Green synthesis of sodium alginate-silver nanoparticles and their antibacterial activity. Int. J. Biol. Macromol..

[67] Obireddy S.R., Bellala S., Chintha M., Sake A., Subbarao S.M.C., Lai W.F. (2023). Synthesis and properties of alginate-based nanoparticles incorporated with different inorganic nanoparticulate modifiers for enhanced encapsulation and controlled release of favipiravir. Arab. J. Chem..

[68] Lemnaru Popa G.M., Trusca R.D., Ilie C.I., Tiplea R.E., Ficai D., Oprea O., Stoica-Guzun A., Ficai A., Ditu L.M. (2020). Antibacterial activity of bacterial cellulose loaded with bacitracin and amoxicillin: In vitro studies. Molecules.

[69] Anghel I., Holban A.M., Grumezescu A.M., Andronescu E., Ficai A., Anghel A.G., Maganu M., Laz R.V., Chifiriuc M.C. (2012). Modified wound dressing with phyto-nanostructured coating to prevent staphylococcal and pseudomonal biofilm development. Nanoscale Res. Lett..

[70] Cotar A.I., Grumezescu A.M., Andronescu E., Voicu G., Ficai A., Ou K.-L., Huang K.-S., Chifiriuc M.C. (2013). Nanotechnological solution for improving the antibiotic efficiency against biofilms developed bygram-negative bacterial strains. Lett. Appl. NanoBioSci..

[71] CLSI (2021). Performance Standards for Antimicrobial Susceptibility Testing.

[72] Motelica L., Ficai D., Ficai A., Trusca R.D., Ilie C.I., Oprea O.C., Andronescu E. (2020). Innovative antimicrobial chitosan/ZnO/Ag NPs/citronella essential oil nanocomposite-potential coating for grapes. Foods.

[73] Spoiala A., Ilie C.I., Trusca R.D., Oprea O.C., Surdu V.A., Vasile B.S., Ficai A., Ficai D., Andronescu E., Ditu L.M. (2021). Zinc oxide nanoparticles for water purification. Materials.

[74] Jovanović Ž., Stojkovska J., Obradović B., Mišković-Stanković V. (2012). Alginate hydrogel microbeads incorporated with Ag nanoparticles obtained by electrochemical method. Mater. Chem. Phys..

[75] Shankar S., Wang L.F., Rhim J.W. (2016). Preparations and characterization of alginate/silver composite films: Effect of types of silver particles. Carbohydr. Polym..

[76] Lozano-Vazquez G., Alvarez-Ramirez J., Lobato-Calleros C., Vernon-Carter E.J., Hernandez-Marin N.Y. (2021). Characterization of corn starch-calcium alginate xerogels by microscopy, thermal, XRD, and FTIR analyses. Starch-Starke.

[77] Motelica L., Ficai D., Oprea O., Ficai A., Trusca R.-D., Andronescu E., Holban A.M. (2021). Biodegradable alginate films with ZnO nanoparticles and citronella essential oil—A novel antimicrobial structure. Pharmaceutics.

[78] Bo S., Luo J., An Q., Xiao Z., Wang H., Cai W., Zhai S., Li Z. (2020). Efficiently selective adsorption of Pb(ii) with functionalized alginate-based adsorbent in batch/column systems: Mechanism and application simulation. J. Clean. Prod..

[79] Li K., Wu G., Wang M., Zhou X., Wang Z. (2018). Efficient removal of lead ions from water by a low-cost alginate-melamine hybrid sorbent. Appl. Sci..

[80] Belalia F., Djelali N.E. (2016). Investigation of swelling/adsorption behavior of calcium alginate beads. Rev. Roum. Chim..

[81] Lopusiewicz L., Macieja S., Sliwinski M., Bartkowiak A., Roy S., Sobolewski P. (2022). Alginate biofunctional films modified with melanin from watermelon seeds and zinc oxide/silver nanoparticles. Materials.

[82] Bibi A., Ur-Rehman S., Akhtar T., Akhter K., Rafique S., Faiz R. (2022). Synthesis of alginate-based nanocomposites: A novel approach to antibacterial films. Chem. Pap..

[83] Zhang F., Gao C., Zhai S.R., An Q.D. (2023). Nanosilver anchored alginate/poly(acrylic acid/acrylamide) double-network hydrogel composites for efficient catalytic degradation of organic dyes. Front. Chem. Sci. Eng..

[84] Kanagaraj S.S.P., Rajaram S.K., Ahamed M., Subedhar S., Sankar K., Innasimuthu G.M., Karuppiah P. (2021). Antimicrobial activity of green synthesized biodegradable alginate–silver (Alg-Ag) nanocomposite films against selected foodborne pathogens. Appl. Nanosci..

[85] Susilowati E., Mahardiani L., Hardini R.D. (2022). The effect of silver nanoparticles toward properties and antibacterial activity of silver-alginate nanocomposite films. Front. Sustain. Food Syst..

[86] Mahapatra A., Padhi N., Mahapatra D., Bhatt M., Sahoo D., Jena S., Dash D., Chayani N. (2015). Study of biofilm in bacteria from water pipelines. J. Clin. Diagn. Res. JCDR.

[87] Maes S., Vackier T., Nguyen Huu S., Heyndrickx M., Steenackers H., Sampers I., Raes K., Verplaetse A., De Reu K. (2019). Occurrence and characterisation of biofilms in drinking water systems of broiler houses. BMC Microbiol..

[88] Vanaja M., Gnanajobitha G., Paulkumar K., RajeshKumar S., Malarkodi C., Annadurai G. (2013). Phytosynthesis of silver nanoparticles by *Cissus quadrangularis*: Influence of physicochemical factors. J. Nanostruct. Chem..

[89] Kedziora A., Wieczorek R., Speruda M., Matolinova I., Goszczynski T.M., Litwin I., Matolin V., Bugla-Ploskonska G. (2021). Comparison of antibacterial mode of action of silver ions and silver nanoformulations with different physico-chemical properties: Experimental and computational studies. Front. Microbiol..

[90] Pavlikova N. (2022). Caffeic acid and diseases-mechanisms of action. Int. J. Mol. Sci..

[91] Khan F., Bamunuarachchi N.I., Tabassum N., Kim Y.M. (2021). Caffeic acid and its derivatives: Antimicrobial drugs toward microbial pathogens. J. Agric. Food Chem..

[92] Lee W.-F., Tsao K.-T. (2010). Effect of silver nanoparticles content on the various properties of nanocomposite hydrogels by in situ polymerization. J. Mater. Sci..

[93] Sharmin N., Pang C., Sone I., Walsh J.L., Fernandez C.G., Sivertsvik M., Fernandez E.N. (2021). Synthesis of sodium alginate-silver nanocomposites using plasma activated water and cold atmospheric plasma treatment. Nanomaterials.

[94] Zakia M., Koo J.M., Kim D., Ji K., Huh P., Yoon J., Yoo S.I. (2020). Development of silver nanoparticle-based hydrogel composites for antimicrobial activity. Green Chem. Lett. Rev..

