# Supplementary material for: The Development of Alginate/Ag NPs/Caffeic Acid Composite Membranes as Adsorbents for Water Purification
Source: Membranes (Basel). 2023 Jun 9;13(6):591. doi: 10.3390/membranes13060591 (PMC10305225; doi:10.3390/membranes13060591)
Supplement: Supplementary file 1 [file membranes-13-00591-s001.zip › membranes-2339337-supplementary.pdf]

membranes

Article

The development of alginate/Ag NPs/caffeic acid composite membranes as adsorbents for water purification

Angela Spoială [1,2], Cornelia-Ioana Ilie [1,2], Georgiana Dolete [1,2], Gabriela Petrișor [1,2], Roxana-Doina Trușcă [1,2], Ludmila Motelica [2,3], Denisa Ficai [2,3], Anton Ficai [1,2,4], Ovidiu-Cristian Oprea [2,3,4]*, Mara-Lia Dițu[5]

[1] Department of Science and Engineering of Oxide Materials and Nanomaterials, Faculty of Chemical Engineering and Biotechnologies, University Politehnica of Bucharest, 1-7 Gh Polizu Street, 011061 Bucharest, Romania; angela.8317@gmail.com (A.S.)
[2] National Centre for Micro and Nanomaterials & National Centre for Food Safety, Faculty of Chemical Engineering and Biotechnologies, University Politehnica of Bucharest, Spl. Indendentei 313, 060042 Bucharest, Romania.
[3] Department of Inorganic Chemistry, Physical Chemistry and Electrochemistry, Faculty of Chemical Engineering and Biotechnologies, University Politehnica of Bucharest, 1-7 Gh Polizu Street, 050054 Bucharest, Romania.
[4] Academy of Romanian Scientists, 3 Ilfov Street, 050045 Bucharest, Romania.
[5] Faculty of Biology, University of Bucharest, 1-3 Aleea Portocalelor, 060101 Bucharest, Romania.
* Correspondence: ovidiu.oprea@upb.ro

Citation: Spoială, A.; Ilie, C-I.; Dolete, G.; Petri, G.; Tru, R-D.; Motelica, L.; Ficai, D.; Ficai, A.; Oprea, O-C.; Di, M-L. The development of alginate/Ag NPs/caffeic acid composite membranes as adsorbents for water purification. *Membranes* **2023**, *13*, 591. https://doi.org/10.3390/membranes13060591

Academic Editors: Smolik Jerzy, Piotr Wieciński and Joanna Kacprzyńska-Gołacka

Received: 28 March 2023
Revised: 28 May 2023
Accepted: 5 June 2023
Published: 9 June 2023

Copyright: © 2023 by the authors. Licensee MDPI, Basel, Switzerland. This article is an open access article distributed under the terms and conditions of the Creative Commons Attribution (CC BY) license (https://creativecommons.org/licenses/by/4.0/).

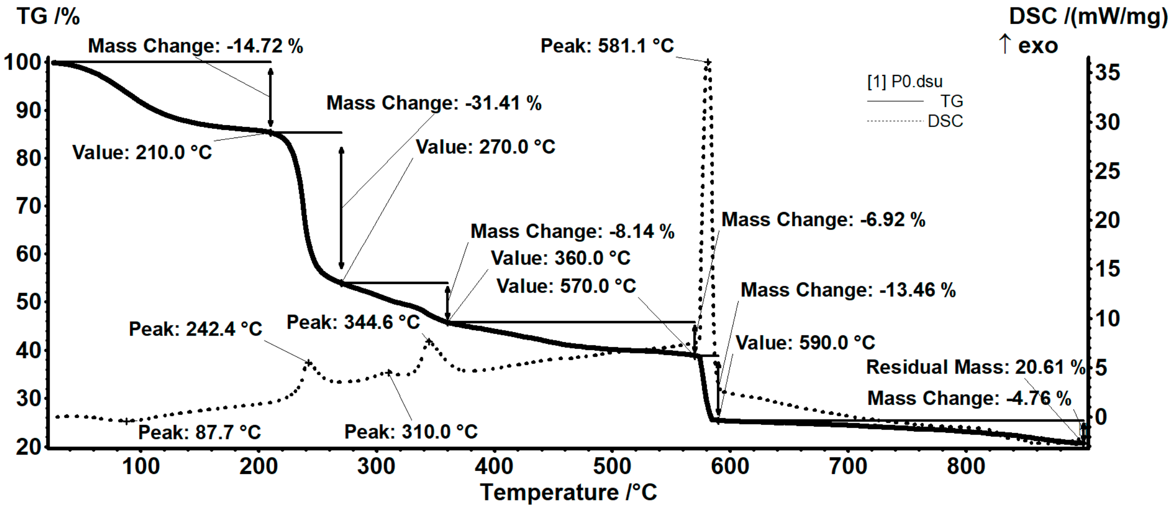

Figure S1. TG-DSC curves of P0 alginate membrane

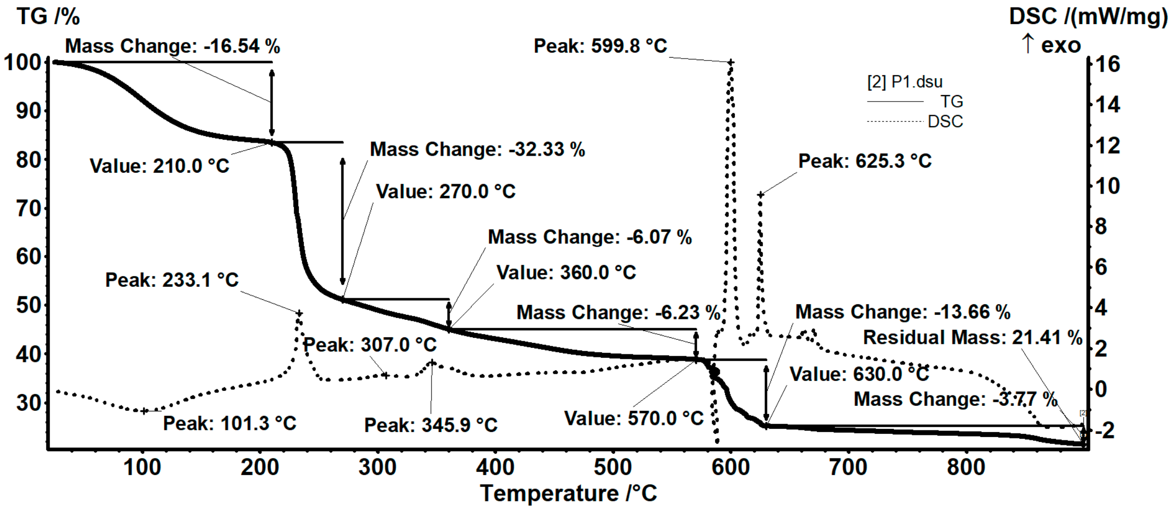

Figure S2. TG-DSC curves of P1 alginate-based composite membrane

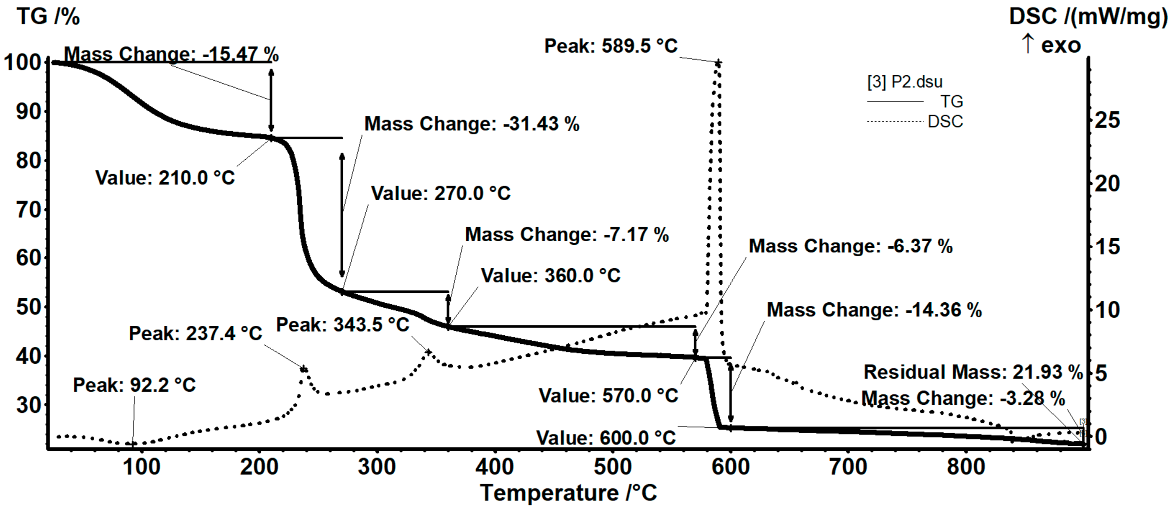

Figure S3. TG-DSC curves of P2 alginate-based composite membrane

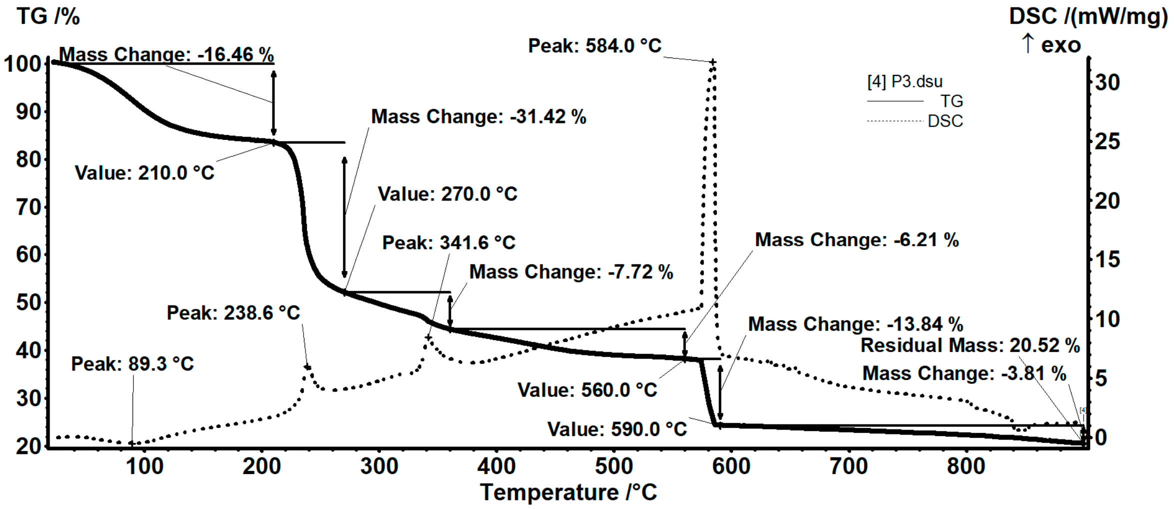

Figure S4. TG-DSC curves of P3 alginate-based composite membrane

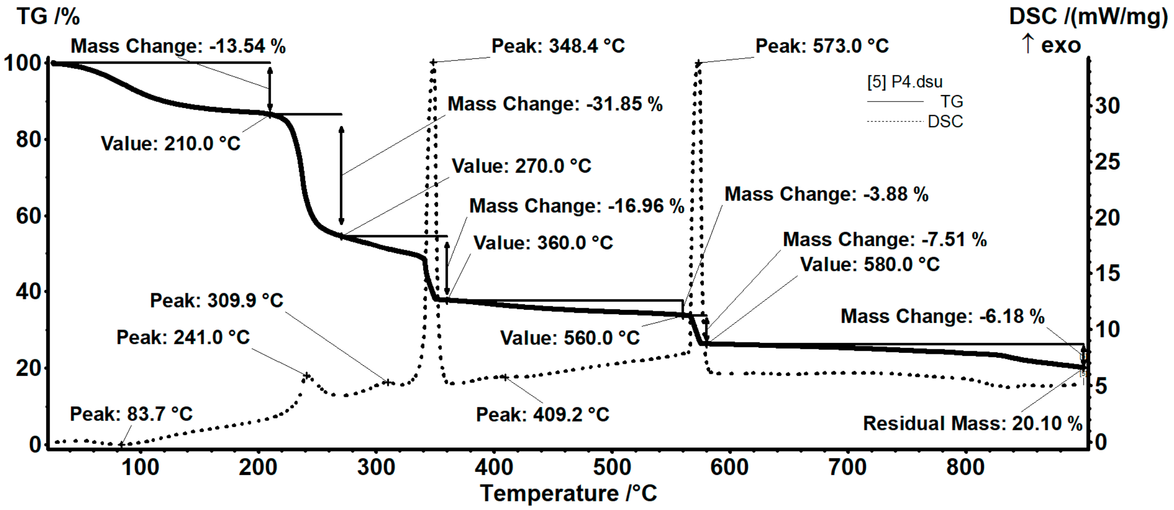

Figure S5. TG-DSC curves of P4 alginate-based composite membrane

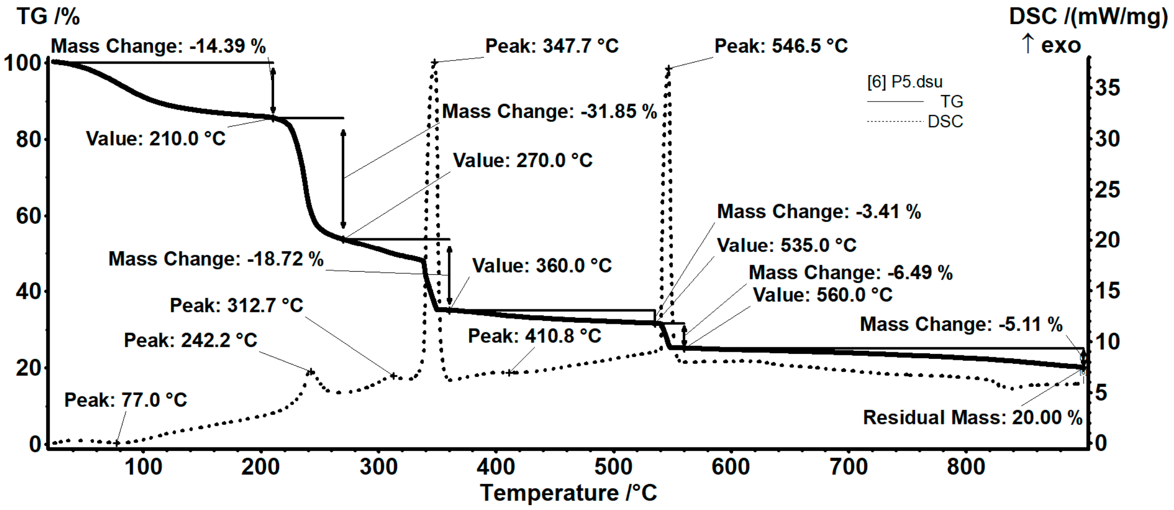

Figure S6. TG-DSC curves of P5 alginate-based composite membrane

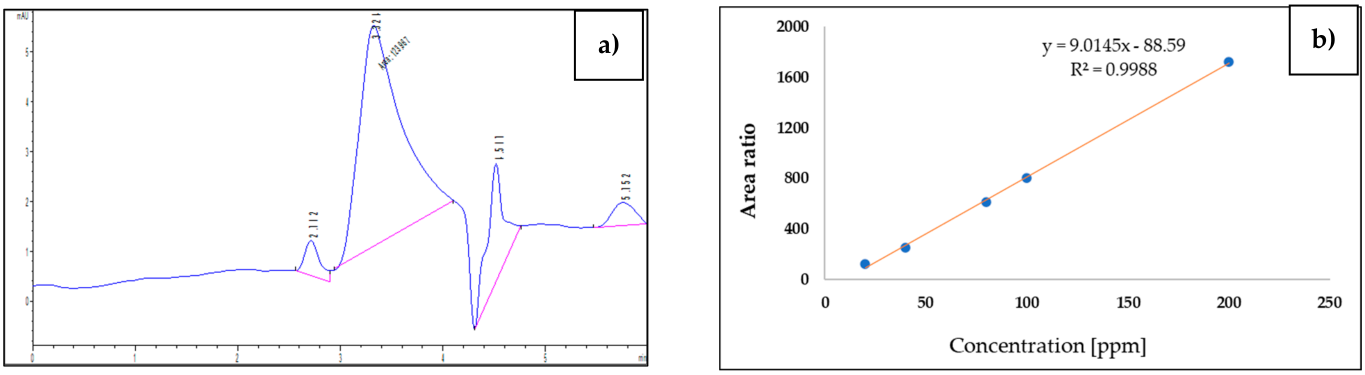

Figure S7. HPLC chromatogram of caffeic acid standards (a) and calibration curve (b)

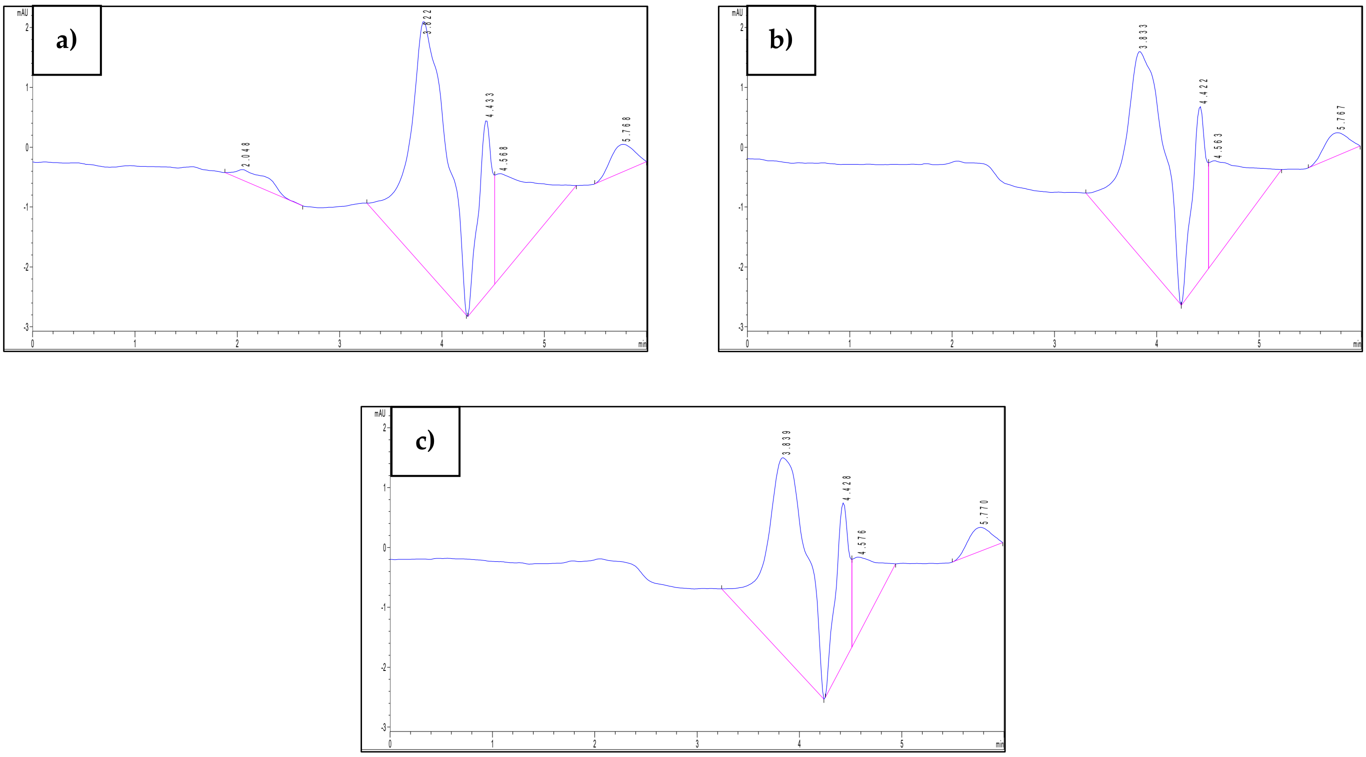

Figure S8. HPLC chromatograms for samples collected after 137h: a) P3; b) P4; c) P5.

Disclaimer/Publisher's Note: The statements, opinions and data contained in all publications are solely those of the individual author(s) and contributor(s) and not of MDPI and/or the editor(s). MDPI and/or the editor(s) disclaim responsibility for any injury to people or property resulting from any ideas, methods, instructions or products referred to in the content.
